# Vibration-induced extra torque during electrically-evoked contractions of the human calf muscles

**DOI:** 10.1186/1743-0003-7-26

**Published:** 2010-06-10

**Authors:** Fernando H Magalhães, André F Kohn

**Affiliations:** 1 Neuroscience Program and Biomedical Engineering Laboratory, Universidade de São Paulo, EPUSP, PTC, Avenida Professor Luciano Gualberto, Travessa 3, n.158, Butanta, São Paulo, SP, Brazil

## Abstract

**Background:**

High-frequency trains of electrical stimulation applied over the lower limb muscles can generate forces higher than would be expected from a peripheral mechanism (i.e. by direct activation of motor axons). This phenomenon is presumably originated within the central nervous system by synaptic input from Ia afferents to motoneurons and is consistent with the development of plateau potentials. The first objective of this work was to investigate if vibration (sinusoidal or random) applied to the Achilles tendon is also able to generate large magnitude extra torques in the triceps surae muscle group. The second objective was to verify if the extra torques that were found were accompanied by increases in motoneuron excitability.

**Methods:**

Subjects (n = 6) were seated on a chair and the right foot was strapped to a pedal attached to a torque meter. The isometric ankle torque was measured in response to different patterns of coupled electrical (20-Hz, rectangular 1-ms pulses) and mechanical stimuli (either 100-Hz sinusoid or gaussian white noise) applied to the triceps surae muscle group. In an additional investigation, M_max _and F-waves were elicited at different times before or after the vibratory stimulation.

**Results:**

The vibratory bursts could generate substantial self-sustained extra torques, either with or without the background 20-Hz electrical stimulation applied simultaneously with the vibration. The extra torque generation was accompanied by increased motoneuron excitability, since an increase in the peak-to-peak amplitude of soleus F waves was observed. The delivery of electrical stimulation following the vibration was essential to keep the maintained extra torques and increased F-waves.

**Conclusions:**

These results show that vibratory stimuli applied with a background electrical stimulation generate considerable force levels (up to about 50% MVC) due to the spinal recruitment of motoneurons. The association of vibration and electrical stimulation could be beneficial for many therapeutic interventions and vibration-based exercise programs. The command for the vibration-induced extra torques presumably activates spinal motoneurons following the size principle, which is a desirable feature for stimulation paradigms.

## Background

Percutaneous electrical stimulation applied directly over the human muscle can elicit contractions by two distinct mechanisms [1,2]: peripheral and/or central. The more common is by the direct stimulation of the terminal branches of motor axons, considered to be of peripheral origin, and hence the generated torque has been called peripheral torque (PT). Alternatively, the stimulation may elicit action potentials in large sensory afferents (favored by the use of low-intensity, wide-pulse-width, high-frequency stimulation [1]) which can synaptically recruit α-motoneurons in the spinal cord. The generated torque has been sometimes called central torque, and has the important feature of being associated with motor unit recruitment in the natural order, starting with the fatigue-resistant units [2-4]. This has obvious beneficial implications for neuromuscular electrical stimulation (NMES), functional electrical stimulation (FES) and other therapeutic interventions. The excitatory input to the motoneurons provided by the sensory volley can produce surprisingly large forces and an unexpected relation between stimulus frequency and evoked contractions [5,6]. For example, when brief periods of high frequency (e.g. 100 Hz) electrical stimulation were delivered on top of a longer train of stimuli kept at a lower frequency (e.g. 25 Hz), there was a large increment in force attributed to the central mechanism. When the stimulation returned to 25 Hz the force remained unexpectedly high [2,5,6]. That is, during a burst-like pattern that alternated periods of 25 and 100 Hz stimulation, more force was generated after the high-frequency burst than before it, despite the similar stimulus frequency and intensity [2,5,6]. In some cases, these sustained forces observed following the high-frequency-bursts could continue even after the end of the stimulation period (i.e. when any stimulus was already turned off) [5].

The "extra force" associated with the central torque, is not present when a nerve block is applied proximal to the stimulation site [5-7], but remains present both in complete spinal cord-injured [5,8] and healthy sleeping subjects [5], which confirms the involuntary and central origin of the phenomenon.

This "extra", self-sustained contraction produced by the involuntary central mechanism, which will be named here "extra torque (ET)", is developed in addition to the torque due to motor axon stimulation [2,5,6,9], and can be quite large, up to 42% of the maximal voluntary contraction (MVC) [6]. Such ET has been proposed to be due to an increase in firing rate and recruitment of new motoneurons through either the development of plateau potentials and/or post-tetanic potentiation (PTP) [5,6]. PTP would increase the release of neurotransmitter from the large sensory axons through high frequency stimulation, thus leading to the activation of higher threshold motoneurons [10]. The sensory volley could also activate motoneuron plateau potentials, trough the opening of voltage-gated L-type Ca ^++ ^channels (for example), thus generating persistent inward currents (PICs) that would produce continuous depolarization (plateau potential) [11-13] and consequently self-sustained motoreuron discharge that may be dissociated from the stimulus pulse [9]

The contraction generated by electrically evoked afferent input to the spinal cord, which is responsible for triggering the ET through a central mechanism, resembles that generated during tonic vibration reflex (TVR), which develops when vibration is applied to a muscle or its tendon. Both mechanisms are triggered by large-diameter afferents, may often outlast the stimulus, develop in a slow fashion and are involuntary but can be abolished by volition [6,14,15]. Furthermore, studies performed in animal preparations have suggested that the activation of plateau potentials also plays a role in the generation of TVR [16].

However, more direct experimental evidence that the firing of human motor units is determined by intrinsic properties such as plateau potentials has been obtained only for a low level voluntary activation of a muscle [17-19]

The present work had as a goal to investigate if vibration is also able to generate large magnitude self sustained ETs, markedly larger than the PT evoked by low-frequency electrical stimulation. More specifically, we aimed to investigate whether vibration may evoke self-sustained forces at levels comparable with those ETs previously shown in response to high-frequency electrical stimulation [2,5,6].

In addition, we sought to investigate if the vibratory stimuli caused an increase in the motoneuron excitability, which could lead to ET from the innervated muscle. In this regard, the F wave is a late response that occurs in a muscle following stimulation of its motor nerve, evoked by antidromic reactivation ("backfiring") of a fraction of the motoneurons and is sensitive to changes in motoneuron excitability [20]. In contrast to the H-reflex, which is dependent on presynaptic inhibition and homosynaptic depression, the F response is not elicited by a Ia volley [21], and would therefore be a useful method for assessing the excitability of the motoneuron pool in this experiment. Although the use of F waves for assessing motoneuron excitability is controversial [21,22], F waves reflect motoneuron excitability in a general way [23].

Finally, it is important to emphasize that there are important differences between the effects of electrical and vibratory stimuli. An obvious difference is the lack of antidromic activation of motoneuron (and sensory) axons during vibration. This means that there is no collision (and annihilation) of reflexively generated action potentials and the antidromic action potentials. In addition, the temporal dispersion of Ia afferent volleys in the tibial nerve induced by Achilles tendon percussion is much greater than that of electrically induced volleys, which may lead to differences in central transmission [24]. Furthermore, group II, Ib and cutaneous afferent discharges induced by electrical stimulation of the tibial nerve are different from those induced by Achilles tendon percussion [25,26]. Hence vibration's ability to evoke extra torques similar to those obtained in response to wide pulse width, high frequency electrical stimulation cannot be easily predicted.

## Methods

### Assessing ET Generation

Six male subjects (30 ± 5.3 (SD) age, ranging from 26 to 37 years) volunteered to participate in this study. The experiments had approval by the local ethics committee and were conducted in accordance with the Declaration of Helsinki. Each subject signed an informed consent document.

Subjects were seated on a customized chair designed for measuring ankle torque during isolated isometric plantarflexion contraction. The hip, knee and ankle of the right leg were maintained at 90° with an adjustable metal bar placed over the anterior distal femur, superior to the patella and fixed to the chair, avoiding any movement of the thigh. The right foot (all subjects were right-footed) was tightly fixed to a rigid metal pedal so that its axis of rotation was aligned with the medial malleolus. A strain gauge force transducer (Transtec N320, Brazil) was attached to the pedal for isometric torque measurements.

At the beginning of the session, each subject's maximal voluntary force during plantarflexion was determined. Subjects were asked to perform three MVCs of the triceps surae (TS), with 2 min rest between each trial. The maximum force value achieved across the three trials was taken as the MVC force value. All measurements in this paper are expressed as a percentage of the MVC (and hence we use the terms torque and force interchangeably).

Flexible silicon stimulating electrodes (10 cm long × 5 cm wide) were fixed over the subjects' right calf muscle. The proximal electrode was positioned midway across the two portions of the gastrocnemius muscles, ~10-15 cm distal to the popliteal fossa. The distal electrode was placed over the soleus, just below the inferior margin of the two heads of the gastrocnemius muscle. A DIA-PULSI 990 stimulator (Quark, Brazil) was driven by a computer that controlled the delivery of rectangular pulses of 1-ms duration. A single burst consisting of 5 pulses at 100 Hz was used in order to set the stimulus intensity, progressively adjusting the current until the peak ankle torque produced by such stimuli reached ~5% of the subject's MVC value [5]. It has been previously demonstrated that such intensity is optimal for generating marked ETs in the TS muscle group in response to burst patterns alternating higher and lower frequencies of electrical stimulation (e.g. 20-100-20 Hz) [2,6].

The Achilles tendon of the right leg was stimulated mechanically by means of a LW-126-13 vibration system (Labworks, USA), consisting of a power amplifier and a shaker (cylindrical body, with diameter 10.5 cm and length 13.5 cm). The shaker was fixed to the bottom structure of the chair, so that the tip of the shaker (round-shaped plastic tip, 1 cm diameter) was pressed against the Achilles tendon in order to keep a steady pressure and a fixed position on the tendon. A LabView system (National Instruments, USA) was utilized to generate either 100-Hz sine waves or gaussian white noise signals with 2-s duration, which were delivered to the input of the shaker's power amplifier in order to obtain the desired mechanical stimulation. An ADXL78 accelerometer (Analog Devices, USA) was attached to the movable part of the shaker in order to monitor the parameters of the mechanical stimuli.

**Figure 1:**
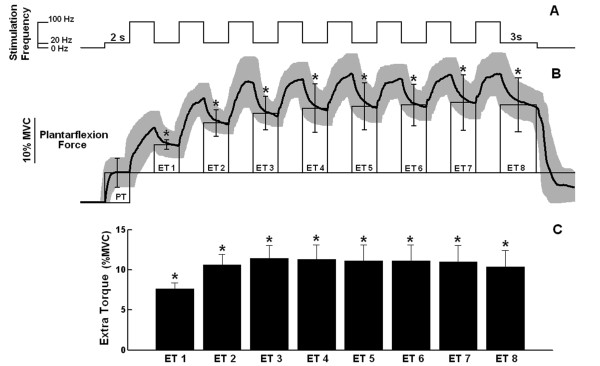
**Peripheral and extra torques generated by *stimulation pattern 1***. **A) **Schematic representation of *stimulation pattern 1 *showing the time course of alternating 2-s of 20-Hz and 100-Hz bursts of electrical stimulation. **B) **Average plantarflexion torque as a function of time (n = 8, thick line) with SD shown in light shade. Bars (thin line) represent the values of peripheral torque (PT) and extra torques (ETs, means ± SDs). Note that the ET values are the increments with respect to the PT value. The eight extra torque values generated by the series of 100-Hz bursts are labeled ET1 -- ET8. Data are from a representative subject. **D) **Average extra torques (± SEMs) representing group data (n = 48). Asterisks indicate extra torque values significantly different from zero (p < 0.05).

**Figure 2:**
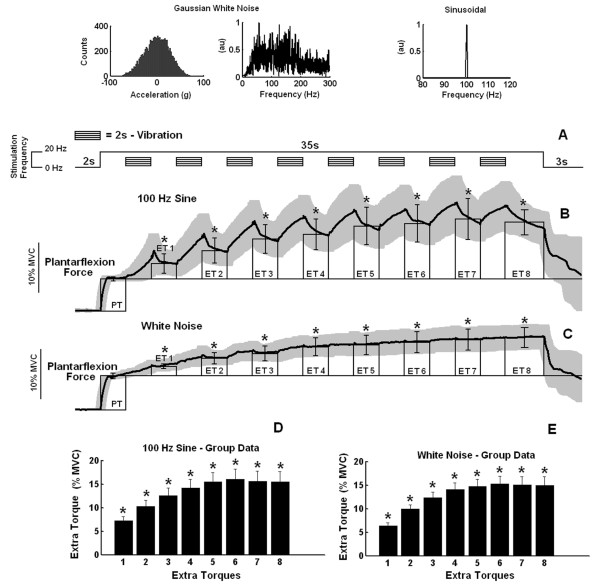
**Peripheral and extra torques generated by *stimulation pattern 2***. At the top, the first two graphs show the amplitude histogram and the absolute value of the FFT of the Gaussian white noise acceleration signal and the third graph shows the absolute value of the FFT of the sinusoidal acceleration signal measured at the tip of the shaker. **A) **Schematic representation of *stimulation pattern *2, showing the time course of 8 intermittent bursts of vibratory stimuli of 2 s duration (rectangular boxes) together with a constant background 20 Hz electrical stimulation. **B) **Average plantarflexion torque as a function of time (n = 8, thick line) with SD shown in light shade. Bars (thin line) represent the values of peripheral torque (PT) and extra torques (ETs, means ± SDs). The eight extra torque values generated by the series of 100-Hz bursts are labeled ET1 -- ET8. Data are from a representative subject. **C) **The same as in B but for the white noise vibratory bursts instead of the 100-Hz sine wave bursts (both B and C are data from the same representative subject). **D and E) **Average extra torques (± SEMs) representing group data (n = 48) for the stimuli utilizing 100 Hz sine waves (D) and white noise (E). Asterisks indicate extra torque values significantly different from zero (p < 0,05).

**Figure 3:**
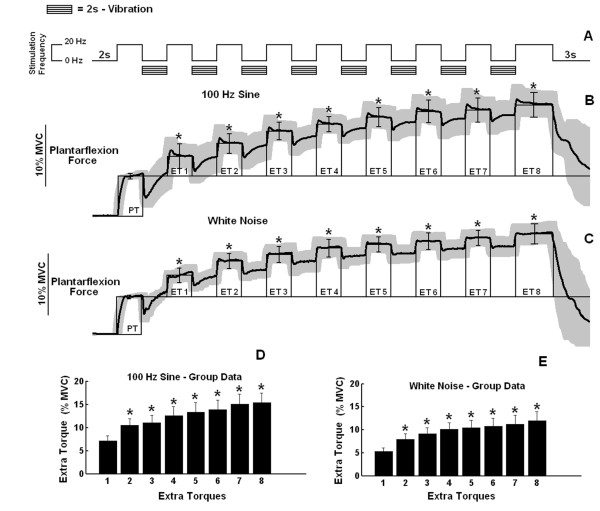
**Peripheral and extra torques generated by *stimulation pattern 3***. **A) **Schematic representation of *stimulation pattern *3 showing the time course of alternated 2 s of electrical and 2 s of mechanical stimuli (rectangular boxes), resulting in 8 bursts of mechanical vibration. **B-E**) The same as in Figure 2, but with data regarding stimulation pattern 3 instead of 2. Data are from the same representative subject from figure 2.

Eight 2-s-bursts of 100-Hz electrical stimulation separated by 2 s of 20-Hz stimulation (starting with a 2-s and ending with a 3-s period of 20-Hz stimulation) were initially applied. Such a pattern (named here *stimulation pattern 1*), is similar to that successfully utilized by previous studies [2,5-7] in order to observe ETs generated by high frequency bursts of electrical stimulation. It is also being included here in order to assure inter-studies repeatability as well to compare, in the same sample of subjects, ETs triggered by electrical stimulation with those triggered by vibration. Additionally, two different patterns of coupled electrical (20 Hz, rectangular 1-ms pulses) and mechanical (either 100-Hz sinusoidal or white gaussian noise pattern) stimulations were utilized, and will be named in the text as stimulation patterns 2 and 3, respectively: 35 s of 20 Hz electrical stimulation together with 8 intermittent bursts of mechanical stimuli of 2 s duration, starting at 2 s and finishing 3 s before the end of the electrical stimuli (stimulation pattern 2); and 35 s of alternated 2 s of electrical and 2 s of mechanical stimuli, resulting in 8 bursts of mechanical vibration (stimulation pattern 3). Thus, 3 different stimulation patterns were utilized, and will be referred in the text as patterns 1 to 3 (see figure 1, figure 2 and figure 3 for examples). In addition, for control purpose, each subject completed two 35 s trials of 20-Hz electrical stimulation.

**Figure 4:**
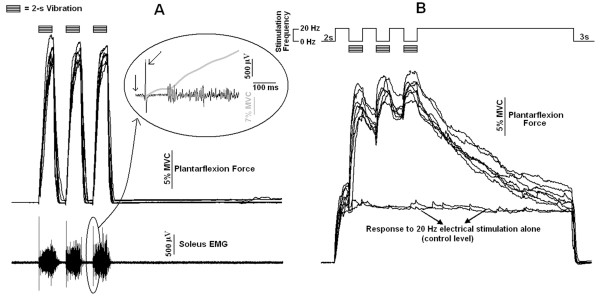
**Responses to three vibratory bursts either alone or alternated with trains of electrical stimulation**. **A) **Plantarflexion torque (seven superposed recordings) and EMG from the soleus muscle (typical recording) in response to three 2-s vibratory bursts (100 Hz sinusoidal waves) separated by 2 s resting periods (no stimulation). The inset of the figure highlights the soleus EMG (black line) and the evoked plantarflexion force (gray line) on an expanded time scale (the two arrows indicate, respectively, the initiation of vibration and the monosynaptic response triggered by the first cycle of the vibratory stimulus). **B) **Plantarflexion force (seven superposed recordings) in response to three 2-s vibratory bursts (100 Hz sinusoidal waves) alternately applied with 20-Hz electrical stimulation (starting with 2s and terminating with 23 s of electrical stimulation). The two approximately constant responses (control values of force) correspond to the plantarflexion force evoked by 37 s of 20 Hz electrical stimulation alone (control stimulation).

In a few subjects, three 2-s bursts of 100-Hz sinusoidal vibration were alternated with 2-s 20 Hz electrical stimulation trains, starting with 2-s and ending with a long train (23 s) of 20 Hz electrical stimuli (see figure 4). Such paradigm was used to evaluate the time decay of the evoked ETs during the last 23 seconds of 20 Hz electrical stimulation alone, as well as to compare its responses with those evoked by TVRs generated by three 2 s of 100 Hz sinusoidal vibration bursts applied without electrical stimuli (see figure 4). These paradigms will be named "additional investigations" in the results section.

When the paradigm involved only vibratory stimulation, the EMG signals from the soleus muscle in response to vibration were acquired simultaneously with the signals from the force transducer and the accelerometer. The EMG signals were amplified and filtered (10 Hz to 1 kHz) by a MEB 4200 system (Nihon-Kohden, Japan). Round-shaped surface electrodes (0.8 cm diameter, proximal-distal orientation, with an inter-electrode distance of 2 cm) were positioned over the soleus muscle, the most proximal contact being 4 cm beneath the inferior margin of the two heads of the gastrocnemius muscle. A ground electrode was placed over the tibia.

The peak-to-peak acceleration of the 100 Hz sinusoidal vibration used in this study was 200.g in the average (200 times the acceleration of gravity). This corresponded to a RMS value around 70.g and a peak-to-peak displacement of the tip of the shaker around 5 mm. The RMS value of the Gaussian white noise vibration was around 27.g (see inset of figure 2 for a visualization of the white noise amplitude distribution and spectrum).

The subjects were asked to relax completely, not making any voluntary effort during the stimulation trials. Each subject completed 8 trials of each stimulation paradigm described above with an inter-trial interval of ~90 s.

A program written in the Workbench environment (DataWave Technologies, USA) was used to deliver trigger pulses in order to synchronize the occurrence of each 2 s of mechanical (sinusoidal or noise) bursts and the start of the torque, EMG and accelerometer data acquisition (sampled at 5 KHz). The same program controlled the pulses delivered by the electrical stimulator.

The evoked forces generated by the stimulation patterns utilized here initially showed a peripheral component, presumably originated from the direct stimulation of motor axons in response to the 20-Hz electrical stimulation. Subsequently, a central component was observed, reflexively evoked from either high frequency electrical stimulation [2,6] or vibration bursts. Finally, the so called ET emerged, defined as the *additional *torque developed over the PT value, triggered by the central mechanism, thus observed *after *the end of a high-frequency electrical stimulation or vibratory burst. The outcome variables of interest in this particular study were the PT and the ET. To quantify them, we adapted a method proposed by Dean and colleagues [2]. PT was defined as the torque level produced during the first 2 s of the 20-Hz-stimulation initially applied (before the delivery of any 100-Hz electrical stimulation or vibration bursts), and ET was quantified as the *additional *torque measured during the following periods of 2 s with no stimuli besides the basal 20 Hz electrical stimulation. To quantify the torque produced during a given time period, the average torque was calculated during the most stable 0.5-s interval contained in that period (i.e. with the smallest coefficient of variation).

### Assessing Motoneuron Excitability

The experiments were performed on three healthy men (30 ± 4.7 (SD) age), with informed consent and the approval of the local ethics committee. These subjects had previously participated in the experiments for assessing ET generation and each had exhibited significant ETs during all the stimulation patterns utilized (see Results). Additionally, these subjects had also shown increased ETs when additional vibratory bursts were delivered (see Results, figure 1, figure 2, figure 3 and figure 4). All procedures and apparatus were identical to those previously described here, except for the stimulation techniques to evoke F waves and the stimulation paradigms employed (i.e. stimulation patterns).

In order to record the M and F waves evoked in response to supramaximal tibial nerve stimulation, the EMG signals from the right soleus muscle were acquired. Round-shaped surface electrodes (0.8 cm diameter, proximal-distal orientation, with an inter-electrode distance of 2 cm) were positioned over the soleus muscle, the most proximal contact being 5 cm below the inferior margin of the two heads of the gastrocnemius muscle (just below the distal silicon stimulating electrode). A ground electrode was placed over the tibia. The EMG signals were filtered from 100 Hz to 1 kHz, the highpass cutoff being chosen higher than usual to attenuate the stimulus artifacts from the 20-Hz percutaneous electrical stimulation.

**Figure 5:**
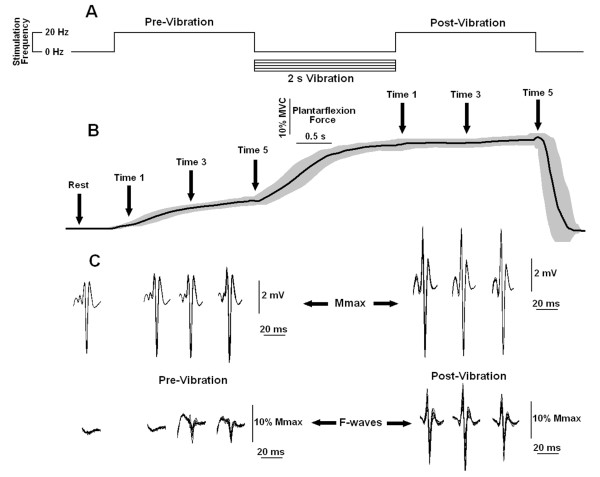
**Output plantarflexion force, M_max _and F-waves generated at rest and during periods of 20 Hz electrical stimulation before and after the delivery of a vibratory burst**. **A) **Schematic representation of a stimulation pattern showing the time course of two trains of 2 s of 20 Hz electrical stimulation separated by a single 2 s burst of vibration (100 Hz sinusoidal waves). **B) **Average torque as a function of time (n = 8, thick line) with SD shown in light shade. The arrows indicate the times (rest, Time 1, Time 3 and Time 5) when the M_max _and F-waves responses shown in (C) were obtained. **C) **M_max _-waves and F - waves recorded from the soleus muscle (10 superimposed repetitions are shown) at the times indicated by the arrows in B. Calibration bars for the M_max _are expressed in mV, while calibration bars for the F-waves are adjusted as a fraction of the corresponding M_max _(i.e. F-waves are normalized to the % of M_max_). Data are from one representative subject.

F waves were evoked by supramaximal electrical stimulation of the posterior tibial nerve (duration, 1 ms) by means of surface electrodes with the cathode (2 cm ^2^) in the popliteal fossa and the anode (8 cm ^2^) against the patella. At the beginning of each session, the maximal peak-to peak amplitude of the soleus compound muscle action potential (maximal M wave, M_max_) was obtained. The stimulus intensity used to elicit F-waves was 180% of that required to elicit the M_max_. A sample of 10 responses were obtained at different times during the stimulation paradigm, both during the initial 2 s of 20-Hz electrical stimulation alone and during the 2 s of 20-Hz electrical stimulation after the delivery of 100-Hz vibratory sine waves stimulation (see figure 5).

One supramaximal stimulus was delivered to the tibial nerve 50 ms after either one of the following pulses of a given burst of 20-Hz percutaneous electrical stimulation applied over the TS: 3 ^rd^, 10 ^th^, 20 ^th^, 30 ^th ^and 40 ^th^. This means that a supramaximal pulse was delivered at one of 5 possible latencies, one chosen at a time, being named here Time1 to Time 5, respectively (see, e.g., figure 5).

In all the cases, stimuli used to evoke the F waves (test stimuli) terminated the stimulation session. That is, no further stimulation occurred after the delivery of a test stimulus. This avoided artifacts from the 20-Hz electrical stimulation to contaminate the signal. Therefore, an independent stimulation trial was performed for each F wave obtained. This ranged from a 200-ms stimulation (test stimulus delivered 50 ms after 3 pulses of percutaneous electrical stimulation at 20 Hz) to a 6.05 s stimulation (test stimulus delivered 50 ms after 2 s of percutaneous electrical stimulation at 20 Hz (40 pulses), preceded by 2 s of percutaneous electrical stimulation followed by 2 s of vibratory bursts).

For control purposes, a sample of 10 responses at rest was also obtained. In addition, F waves were also obtained in response to a 2-s vibratory burst applied to the Achilles tendon alone (i.e. with no concomitant percutaneous electrical stimulation). For this, test stimuli (n = 10) were delivered to the tibial nerve 200, 550, and 1050 ms after the vibration (analogous to Time1 to Time 3).

### Statistical Analysis

An Analysis of Variance (ANOVA) with repeated measures and Bonferroni's post hoc tests (the latter performed where any significant main effects was pointed out by the preceding ANOVA test) were used to test whether each stimulation paradigm produced significant ETs and whether ETs differed from each other, both within single subjects and group data. Contrasts were performed at a 0.05 level of significance and ET was considered to be significant when it was significantly greater than zero [2] (i.e., when the total torque value taken after each burst of high-frequency electrical or vibratory stimulation was significantly greater than that generated by the peripheral mechanism). All the analyses were performed using the statistical package SPSS 15.0 for Windows (SPSS, Inc., Chicago, Illinois).

A descriptive analysis was used for the data regarding the F wave experiments. This was so because a sample of 3 subjects is not large enough for quantitative statistical tests.

## Results

### Stimulation Pattern 1

Stimulation pattern 1, which alternated between 2-s-bursts of low frequency (20 Hz) and high frequency (100 Hz) percutaneous electrical stimulation (see above), generated significant ETs (figure 1) in all the six subjects examined. The first high frequency burst was sufficient to evoke a significant ET. However, when additional bursts were delivered, two distinct responses could be observed: (1) in half of the subjects, a further increase in ET could be achieved by the subsequent 100 Hz bursts, until a plateau was reached by the third or fourth bursts (see figure 1B for example); the group data (6 subjects, 48 trials) showed the same behaviour (figure 1C); and (2) in the remaining three subjects, a significant decrease in torque was observed after the second or third bursts, i.e., the last five or six high frequency stimulation bursts were not able to generate significant ETs (i.e., not significantly different from zero). This adds further information to previous studies [2,9] that reported, in healthy populations, that some subjects do not generate any ET in response to wide-pulse electrical stimulation. Here, although all subjects were able to generate significant ET at the beginning of the stimulation, some of them could not maintain the extra force after the delivery of each high-frequency burst.

### Stimulation Pattern 2

In all subjects, a significant ET could be observed after the first 100-Hz burst of the vibratory pattern was applied to the Achilles tendon (during stimulation pattern 2) (see figure 2). Additional sinusoidal vibration bursts further increased ET values in four of the six subjects, achieving a steady value by the fourth or fifth bursts (figure 2B, for example). Again, this finding occurred also for group data (figure 2D, 6 subjects, 48 trials). In the other two subjects, the ET evoked by the first vibration burst either remained unchanged along the next 8 bursts or dropped to values not significantly different from zero after the fourth burst.

Similarly, the first burst of the mechanical noise pattern applied to the Achilles tendon was sufficient to evoke significant ET in all subjects during stimulation pattern 2 (see figure 2) and the subsequent mechanical noise bursts increased ET further, until it reached a steady value by the fourth or fifth bursts. The group data followed this same behaviour (figure 2E). In two of the subjects (the same as before), a slight decrease in torque could be observed starting at the fifth or sixth bursts, but such a decrease was not significant.

### Stimulation Pattern 3

When the electrical stimulation was turned off during the application of the vibratory bursts (stimulation pattern 3), significant ETs could be observed in four of the six subjects examined, for both sinusoidal and white noise patterns, reaching a steady value around the fifth burst (figure 3B and 3C). This was similarly found for the group data, ETs achieving significance starting at the second vibratory burst (figure 3D and 3E). For the remaining two subjects, such stimulation did not produce significant ETs.

### Additional Investigations

An example of three TVRs generated in response to three 2-s vibratory bursts (composed of sinusoidal waves) separated by 2-s resting periods (no stimulation) is illustrated in figure 4A. The upper signals (7 trials, 1 subject) show the evoked plantarflexion force waveforms and the lower signal shows the soleus EMG activity corresponding to one of the trials. The inclined arrow in the inset shows a single large EMG response at ~45 ms after the onset of the vibration, probably corresponding to the monosynaptic reflex triggered by the first cycle of the vibratory stimulus. After a silent period of ~100 ms, the EMG activity began to gradually build up simultaneously to an increase in plantarflexion torque (gray curve), characterizing the slow development of the TVR. After the stimulation pattern ended, torque and EMG promptly returned to pre-stimulus levels, as they also did between the vibration bursts. When three bursts of 100-Hz sinusoidal vibration were alternately applied with 20-Hz electrical stimulation (figure 4B), the force exerted by the TS increased during the vibratory stimuli to levels comparable to those achieved by vibration alone. However, after the end of each vibratory burst, the plantarflexion force did not fall promptly to the control level (nearly constant responses in figure 4B). The force signal continued at high levels long after the vibratory bursts were turned off, gradually decreasing to the control values associated with the 20-Hz electrical stimulation.

### Motoneuron Excitability (M_max _and F waves data)

**Figure 6:**
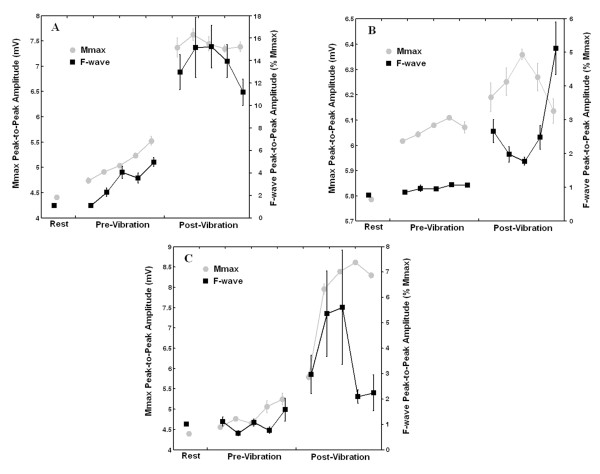
**M_max _and F-wave amplitudes measured at rest and during periods of 20 Hz electrical stimulation before and after the delivery of a vibratory burst**. Peak-to-peak amplitude (n = 10, ± SEM) of the F-waves (black squares, expressed in the right axis as % of M_max_) and the M_max _responses (light gray circles, expressed in the left axis in mV) obtained at rest and at Time 1 to Time 5, both before and after the delivery of the 2 s vibratory burst (100 Hz sinusoidal waves). Note that during both the pre-vibration and the post-vibration phases the 20 Hz electrical stimulus train is being applied (see figure 5A). A, B and C are data taken from the three different subjects.

At different times (Time 1 to Time 5) during the 20 Hz electrical stimulation, the F waves and M_max _evoked after the delivery of the vibratory bursts showed peak-to-peak amplitudes larger than those obtained before vibration (figure 5 and figure 6).

**Figure 7:**
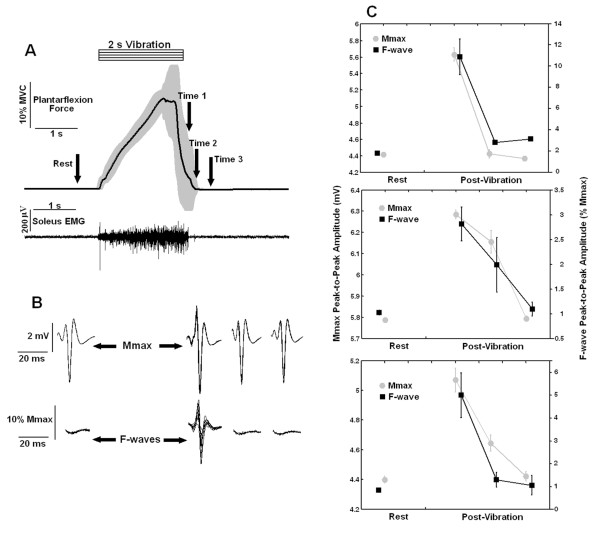
**Output plantarflexion force, soleus EMG, M_max _and F-waves generated at rest and after the delivery of a single vibratory burst**. **A) **Average plantarflexion torque as a function of time (n = 8, thick line, with SD shown in light shade) and EMG from the soleus muscle (from one of the subjects) in response to a single burst of vibration (2 s of 100 Hz sinusoidal waves). The arrows indicate the times (rest, Time 1, Time 2 and Time 3) at which the M_max _and F-waves shown in (B) were obtained. **B) **F-waves and M_max _recorded from the soleus muscle (10 superimposed repetitions are shown and data were taken from a representative subject) at different times after the vibration (Time 1, Time 2 and Time 3) and at rest. **C) **Peak-to-peak amplitude (n = 10, ± SEM) of the F-waves (black squares, expressed in the right axis as % of M_max_) and the M_max _responses (light gray circles, expressed in the left axis in mV) obtained at rest and at Time 1 to Time 3 after the vibration was turned off (as represented in A). Data are from three different subjects.

After the delivery of a 2s vibratory burst alone (i.e, without the 20 Hz electrical stimulation), torque and EMG promptly returned to pre-stimulus levels (figure 7), similar to the responses observed in figure 4. Soon after the end of the vibration (i.e., at Time 1, 200 ms after vibration ended), clear increases in the peak-to-peak amplitudes of F waves and M_max _were observed (figure 7B and 7C). However, such increases did not persist (as they did when alternated with the 20 Hz electrical stimulation, figures 5 and figure 6), but returned to the control levels already at Time 2 or Time 3 (figure 7B and 7C).

## Discussion

The results showed that vibration bursts (either high frequency sinusoids or white noise) delivered to the Achilles tendon can consistently increase the force generated by the TS muscle group while a basal train of 20-Hz electrical stimuli is applied to the TS. In most of the subjects, the vibratory bursts were able to keep the increased force even when the electrical stimulation was turned off during the vibration (alternating vibration with electrical stimulation). An additional investigation showed that the ET generation was accompanied by an increase in the amplitude of the F waves evoked in response to supramaximal tibial nerve stimulation. The paradigm employed here involved no basal voluntary contraction and the ETs triggered by the central mechanism were of substantial amplitude. To our knowledge, this study presents the first direct demonstration that markedly increased ETs, reaching values up to 50% MVC in different subjects, can be triggered reflexively by vibratory stimuli. In average, such increments were 180% of the PT value, ranging from no increment up to a nine-fold increase in torque over the PT value, in different subjects. Both presynaptic (PTP) and postsynaptic (PICs) mechanisms may contribute to these findings, due to the high frequency activation of large sensory afferents from the muscle spindles [27].

The experiments showed that vibratory bursts can generate ETs at levels comparable with those additional forces triggered in response to high-frequency electrical stimulation (see figure 1C, figure 2D, figure 2E, figure 3D and figure 3E). Extra torques could be generated either with or without a continuous background 20-Hz electrical stimulation applied simultaneously to the vibratory bursts (figure 2 and figure 3). When the electrical stimulation was turned off during vibration (in stimulation pattern 3, figure 3), the vibratory bursts caused a torque-interpolation by keeping on the mechanism for extra force generation. From an engineering point of view, the behaviour of the torque signals (compare figure 2 and figure 4) show that the two inputs (an electrical stimulus train and the intermittent vibratory bursts) combine in a nonlinear way to generate the output torque as a function of time. The probable mechanisms are dealt with in the text ahead, but from an input-output point of view, the results indicate the importance of mixing the electrical stimulation (either basal or alternating) with the intermittent vibratory input to secure a change in the dynamics of the system and hence be able to obtain increased torque levels.

The results of the current study are an extension of previous reports [1,2,5,6,8,9] that suggested a central mechanism contributing to extra torque generation when surface NMES was applied to the subject's leg (with similarities to stimulation pattern 1 used in this study). In the new paradigms, the interpretations are perhaps simpler than in the NMES experiments of previous reports [1,2,5,6,8,9] because no antidromic activation of motoneuron axons occurs during the vibratory stimulation as may happen for electrical stimulation. In addition, the vibratory stimulation may induce motoneuron discharges in synchrony with the stimulus [27,28] which does not happen during high-frequency tetanic electrical stimulation [28], probably due to differences in the size of the evoked afferent volley [10].

Gorassini and colleagues [17] showed evidence of self-sustained firing in motoneurons of the intact human as vibration of the tibialis anterior muscle recruited an additional motor unit, beyond the one that was already firing due to the maintenance of a low level background voluntary contraction (< 10% MVC). The recruitment of this second motor unit caused an average sustained increase in the associated dorsiflexion force of 2% of the background force value (their figures 1a and figure 2b). Other studies have also shown that the tonic vibration reflex (TVR) can evoke self-sustained motor unit firing patterns in healthy subjects [18,19,29], with the development of a concurrent low-magnitude force increment. In a recent report, McPherson and colleagues [30] showed an increased TVR response in addition to a sustained electromyographic activity and torque generation (< 1% MVC) after vibration cessation in the paretic upper limb compared with the non-paretic one of individuals with chronic hemiparetic stroke, suggesting that PICs contribute to the expression of altered reflexes following stroke. However, the concurrent increment in force generation associated with the additional motoneuron firing described in the papers above was of very low magnitude. This was due to limitations imposed by the experimental paradigms involved, since single motor unit firing must be assessed while a low-level voluntary contraction is performed. In comparison with these reports that dealt with low magnitude forces, our study showed large increments in plantarflexion force induced by the vibratory bursts (e.g., up to 50% MVC).

During the F wave study, a clear increase of the peak-to-peak amplitudes of the M_max _was observed, both for the responses obtained during the first 2 s of 20 Hz electrical stimulation compared to rest and for the responses obtained during the 2 s of 20 Hz electrical stimulation after vibration compared to those obtained during the first 2 s of 20 Hz electrical stimulation before vibration (figures 5C and figure 6). This is in agreement with recent data [31] that showed substantial increases on the M_max _amplitudes with increasing levels of voluntary contraction of the soleus muscle, even though the ankle position (joint angle) remained unchanged. This shows that compound muscle action potentials (such as M_max _and F waves) can be influenced by peripheral factors at the recording site [31], supporting previous recommendation [32] that in reflex studies it is necessary to normalize M wave and reflex response amplitudes to the corresponding M_max _obtained at the same joint angle and under the same experimental conditions. Using such a normalization procedure, the present study showed a clear increase of the peak-to-peak amplitudes of the F waves for the responses obtained during the 2 s of 20 Hz electrical stimulation after vibration compared to those obtained during the first 2 s of 20 Hz electrical stimulation before vibration. Thus, it is suggested that motoneuron excitability is increased in a general way [23] during a 20 Hz electrical stimulation applied after the delivery of brief vibratory bursts. The increased excitability persisted during the whole time course of 20 Hz electrical stimulation (2 s) delivered after vibration.

The facilitation found at the level of the motoneuronal pool in the experiments with vibration occurred despite the possible development of presynaptic inhibition caused by the vibratory bursts. The vibration-induced presynaptic inhibition takes some time to build up and decays in a few hundred milliseconds [33], therefore it could affect somehow the quantified ETs, although probably not along its whole time course (2 s). Furthermore, successive activation of the Ia afferents could lead to postactivation synaptic depression [10], which would certainly outlast the 2 s interval. However, a more refined analysis in cats has shown that the EPSP amplitude modulation depends on the type of motoneurons analyzed [4]: high threshold motoneurons (associated with fast motor units) were found to have synapses from the Ia afferents that do not depress or may even facilitate for high frequency stimulation. Data from humans have suggested that synapses from Ia afferents depress less in higher threshold motoneurons [34]. In addition, afferents other than the Ia type could also exert a role in the generation of the response to the vibratory bursts [35,36]. Muscle spindle secondary endings as well as Ib tendon afferents could also respond to either sinusoidal or white noise vibration, even if not in a 1:1 relationship with each cycle [36]. Also, recurrent inhibition from Renshaw cells may be involved, since the motoneurons may be recruited in synchrony with the sinusoidal vibration [27] or with peaks of the noise vibration burst. Thus, inhibitory effects to the TS motoneuronal pool (mainly by Ib afferents and possibly by postactivation depression, presynaptic and recurrent inhibition) could have exerted a role, which could explain why significant ETs could not be observed in a few subjects or could not be sustained.

We propose that the neural mechanisms behind the vibration-induced ETs shown here are probably analogous to those previously suggested for electrical stimulation patterns using wide pulse-widths [2,5,6]. Primary muscle spindle ending responses to muscle vibration (ether sinusoidal or white noise) would lead to repeated activation of large Ia sensory afferents resulting in PTP [10] (a presynaptic mechanism). In addition, the excitatory input provided by the sensory volley could lead to the development of plateau potentials in the motoneurons (a postsynaptic mechanism). A transient depolarization of sufficient amplitude and duration ("on" stimulus) can initiate a plateau potential [37], as it would be the case of TVRs evoked by the vibratory bursts in this investigation.

The substantial increment in the F wave amplitudes observed in the present work is clear evidence that motoneuron excitability is higher during the 20 Hz electrical stimulation following vibratory bursts than during the 20 Hz electrical stimulation before the vibration.

The findings (e.g., figure 2B, figure 3B and figure 3C) that in many cases the quantified ETs became more prominent as additional vibratory bursts were delivered is consistent with the "wind up" phenomenon previously reported both in humans and animal preparations [38]. A gradual increase in neurotransmitter release (by PTP) could lead to the development of plateau potentials in additional motoneurons [2] enhancing the increase in the excitability of the motoneuron pool and facilitating the genesis of bistable behavior.

The mechanism for plateau potential generation postulated here as occurring in the motoneurons may also have been originated at a premotoneuronal level. That is, the possibility of plateau potentials to be generated in interneurons within the spinal cord cannot be neglected [6,39]. Therefore, the sustained muscle contractions induced in this study may have been maintained by autonomous activity of motoneurons and/or interneurons in the spinal circuits.

A great inter-subject variability both in the waveforms generated in response to the stimulation paradigms (compare figure 3 and figure 4B) and in extra torque values were observed (CV = 81%). Similarly, previous studies have reported high variability in extra torques elicited through electrical stimulation [2,6]. They could be attributed to inter-subject variations in the levels of monoamines such as serotonin and norepinephrine within the spinal cord, known to be related with the development of PICs in animal studies [40,41]. Other factors that were not controlled in our study can affect the presence of self-sustained motoneuron firing, such as caffeine intake [29]. In addition, different time course and magnitude of PTP between subjects could have accounted for this great inter-subject variability in extra torques.

Although the subjects were asked to relax completely, the possibility of a supraspinal contribution to our results cannot be excluded. For example, it has been shown that electrical stimulation may induce changes in cortical excitability [42], a question not addressed here. However, previous studies using burst patterns of NMES, similar to stimulation pattern 1 in this study [5], have suggested that a voluntary drive to the motoneuron pool is not necessary, as additional forces also emerge in sleeping subjects and in patients with spinal cord transection [8], a finding also consistent with motor unit recordings in both spinal cord-injured humans [43] and rats [44].

In addition, the absence of voluntary contractions in our study makes it less likely that intrafusal thixotropy plays a role [45]. However, such an influence cannot be discarded, as it seems that preconditioning vibration may enhance subsequent TVRs, consistent with the development of intrafusal thixotropy [46]. In this line, a possible influence of other peripheral mechanisms such as extrafusal thixotropy and muscle potentiation from myosin light chain phosphorylation cannot be excluded as well. But, even if these other mechanisms contribute to the effects seen in the generation of ET, there is a clear contribution from a central component, as shown here by means of the F wave.

The concurrent low-frequency electrical stimulation was essential to make the extra torques induced by vibration observable. When the same level of sinusoidal vibration stimulus was applied without the following 20-Hz electrical stimulation, the force promptly returned to the pre-stimulus level after vibration cessation. On the other hand, self-sustained extra forces could be observed when the vibratory bursts were alternated with the 20-Hz electrical stimulation (figure 4). The force waveform in this situation was quite different from that without the background 20 Hz electrical stimulation, being much smoother and outlasting the stimulation by several seconds. Similarly, increased motoneuron excitability (as evidenced by an increase on the F waves amplitude) was observed when the vibratory bursts were followed by the 20 Hz electrical stimulation (figures 5 and 6). When such electrical stimulation was not delivered, an increased excitability was evidenced soon after the vibration was applied alone (200 or 550 ms after, figure 7), but this higher excitability could not be sustained as it was when the vibration was followed by 20 Hz electrical stimulation. Without the following electrical stimulation, the motoneuron excitability evidenced by an increase on the F waves amplitude quickly dropped to levels similar to those observed at rest (figure 7).

Overall, the data presented in this study has shown that, in most subjects, the combination of brief (but powerful) vibratory bursts applied to the tendon of the TS and percutaneous electrical stimulation to the same muscle group can evoke extra self-sustained forces of considerable magnitude. This adds further evidence that intrinsic mechanisms such as plateau potentials may play an important role in regulating the firing of human motor units, which can be intrinsically maintained, reducing the need for prolonged synaptic input, assisting in sustaining contractions during daily activities such as voluntary movements or postural tasks [17]. Proprioceptive drive from muscle spindles is certainly one of the excitatory inputs underlying the development of motoneuronal PICs.

### Practical Relevance

NMES is a widespread tool used in a large diversity of rehabilitation protocols. In addition, FES produces muscle contractions that may result in functional movements in individuals with spinal or supraspinal lesions [47]. However, the conventional stimulation paradigms used to produce muscle force mainly stimulate the terminal branches of motor axons, resulting in a faster development of fatigue [48]. This is so, because motor units are recruited in a random order or with the fast fatigue muscle fibers being activated first [49] (i.e., in the opposite order that occurs during voluntary contraction), which results in a greater metabolic demand relative to the force that is evoked [50]. Consequently, the rapid development of fatigue has been one of the factors limiting the clinical and training effectiveness of NMES and FES [50-52].

Here, we showed that brief vibration bursts (either sinusoids or white noise) delivered to the Achilles tendon could consistently increase the force generated by the triceps surae (TS) muscle group of able-bodied subjects while a basal train of electrical stimuli (20 Hz) was applied to the TS. As the command for such extra force originated within the central nervous system, the resulting activation of spinal motoneurons would follow the size principle, i.e., with fatigue-resistant motor units recruited first. This would be beneficial for therapeutic interventions designed to decrease muscle atrophy (in which the primary cause is the disuse-related loss of fatigue-resistant fibers [53]), or in rehabilitation protocols after spinal cord injury (in which paralyzed muscles often become more easily fatigued [54,55]). As an aside, the recruitment of motor units in their natural order may also be beneficial for training regimes involving the use of NMES in order to improve muscle performance.

Furthermore, substantial increases have been described in the myoelectrical activity of various muscles after 4-5 weeks of training by NEMS, a time not sufficient to induce muscle hypertrophy [56,57]. This has led to the suggestion that certain types of NMES may induce adaptations within the neural systems [58], a hypothesis strengthened by the observation that short NMES training programs may cause an enhancement or diminishment in motor activity of the non-exercised contralateral limb [59]. We also suggest that the underlying mechanisms of neuronal adaptations may be optimised by the use of stimulations techniques that favour the stimulation of sensory axons, leading to enhanced contractions mediated by a central mechanism, as obtained by the combination of vibratory and electrical stimulation.

Significant extra forces were centrally triggered in response to either 100-Hz sinusoidal or white noise vibration (see inset of figure 2 for further details about the white noise characteristics). The latter had the advantage of requiring a lower intensity vibratory stimulus (RMS= ~27.g) than the former (RMS = 70.g). This improved efficiency may arise because the white noise vibration (power spectrum mainly concentrated between 30 and 200 Hz) may stimulate with similar effectiveness type Ia and II spindle afferents besides other mechanoreceptors. From a practical standpoint, this means that the vibratory bursts used to induce extra torque may be weaker than those required by the sinusoidal vibration used in this study (peak-to-peak displacement of the tip of the shaker around 5 mm, or, peak-to-peak acceleration of 200.g), and less specific than a 100 Hz stimulus. This raises the possibility of activating such extra forces by less specialized vibration devices, therefore, making the technique a useful tool in clinical practice.

An issue that has recently been widely discussed concerns the effect of vibration and exercise on human performance. Exercise protocols in association with whole-body vibration [60] or vibrating specific body regions [61] has been used in able-bodied individuals, subjects with pathologies [62] and athletes [63,64] in order to improve muscle force, resistance to fatigue and neuromuscular control. However, despite its appeal, the real effectiveness of vibration and the physiological mechanisms involved in the adaptive responses to vibration exercise are still controversial [63]. The present results suggest that vibration associated with electrical stimulation may provide an effective means of improving human muscle performance, since the electrical stimulation was shown here to be essential to "turn on" the vibration-induced extra torques.

A clear advantage of obtaining extra torque in response to vibration on the electrically stimulated contracting muscle is that separate stimulus sources (i.e. mechanical and electrical) are used. For example, combining different patterns of electrical stimulation like alternated trains of high and lower frequencies (as done, e.g, in [1,2,5-7]) is not useful from a practical point of view, since it requires a sophisticated control of the stimulation, which is not feasible with the conventional stimulators usually employed in clinical and training practice. Therefore, triggering the mechanism for extra torque generation by a separate stimulus source that is commonly used in clinical practice (i.e. vibration) would be helpful.

### Future Directions

The stimulation paradigms employed in this study were designed in order to demonstrate the feasibility of obtaining large extra torques in response to vibratory bursts combined with electrical stimulation. However, from a practical standpoint, future research must be carried out in order to further explore the most suitable parameters of coupled mechanical and electrical stimuli in order to obtain optimized levels of force and improved smoothness of force output. The best way of stimulation will be different for physical therapy/rehabilitation and physical training, as the latter usually employs lower frequency vibratory stimuli. In this line, adjustable forms of stimulation (e.g. persistent random or sinusoidal vibration *versus *vibratory bursts, pairs of parameter values of the vibration and electrical stimuli, sites of vibration application, electrical stimulation parameters, etc.) should be tested, seeking the most adequate one to be utilized for different clinical and practical purposes.

## Conclusions

These results showed that the combination of brief vibratory bursts applied to the tendon of the TS and percutaneous electrical stimulation to the same muscle group can evoke extra self-sustained forces of considerable magnitude. A parallel increase in F-wave amplitudes provided evidence that intrinsic mechanisms such as plateau potentials may play an important role in modulating the firing of human motor units, reducing the need for prolonged synaptic input.

The association between vibration and electrical stimulation could be beneficial for many therapeutic interventions and vibration-based exercise programs because of the increased efficiency, i.e., a larger and more prolonged torque development. As it is reflexively generated, it is less fatiguing because the motor units are recruited following the size principle.

## Abbreviations

ANOVA: analysis of variance; EMG: electromyogram or electromyography; ET: extra torque; FES: functional electrical stimulation; MVC: maximal voluntary contraction; NMES: neuromuscular electrical stimulation; PIC: persistent inward current; PT: peripheral torque; PTP: post-tetanic potentiation; SD: standard deviaton; TS: triceps surae; TVR: tonic vibration reflex.

## Competing interests

The authors declare that they have no competing interests.

## Authors' contributions

Both authors were equally involved in the conceptualization and design of the study. FHM recruited subjects, managed data collection, completed data analysis and drafted the manuscript. AFK supervised data collection, assisted with drafting and provided critical revision of the manuscript. Both authors read and approved the final manuscript.
